# Epi-liquidomics: redefining cancer diagnostics through epigenetic profiling

**DOI:** 10.3389/freae.2026.1813822

**Published:** 2026-04-10

**Authors:** Shahper Nazeer Khan, Mohammad Harris Siddiqui, Aamir Ahmad, Luca Guarnera, Salamatu Tijjani Isah

**Affiliations:** 1 Integral Centre of Excellence for Interdisciplinary Research (ICEIR), Integral University, Lucknow, India; 2 Department of Biosciences, Integral University, Lucknow, India; 3 Department of Bioengineering, Integral University, Lucknow, India; 4 Hamad Medical Corporation, Doha, Qatar; 5 Policlinico Tor Vergata, Rome, Italy

**Keywords:** circulating cell-free DNA (cfDNA), epigenetic profiling, epi-liquidomics, healthcare, liquid biopsy, precision medicine

## Abstract

Early detection is central to reducing cancer related morbidity and mortality, as timely intervention significantly improves curative potential. However, conventional diagnostic modalities such as imaging and tissue biopsy often lack sensitivity for early-stage disease and are inherently invasive. To address these limitations, we introduce Epi-Liquidomics, defined as the comprehensive profiling of circulating epigenetic alterations including DNA methylation patterns, histone derived nucleosome signatures, chromatin fragmentation landscapes and non-coding RNA cargo to enable non-invasive cancer diagnostics supported by advancing technologies. Emerging preclinical and clinical evidence indicates that tumor associated DNA methylation and cfDNA fragmentation signatures are detectable even in stage I–II cancers, directly supporting their applicability in early disease identification rather than solely conceptual potential. By integrating multi-layered epigenetic signals into liquid biopsy platforms, Epi-Liquidomics enhances cancer detection in low tumor-fraction settings and enables accurate tissue-of-origin prediction, offering advantages over mutation centric genomic assays. Beyond early diagnosis, Epi-Liquidomics provides insights into tumor biology by capturing dynamic regulatory states and microenvironment-associated alterations, facilitating improved risk stratification and longitudinal disease monitoring. This framework supports real-time assessment of therapeutic response and detection of minimal residual disease. Importantly, its current limitations including false-positive rates in population screening, assay standardization, and biological variability underscore the need for rigorous prospective validation before routine clinical implementation. As sequencing technologies and computational tools continue to advance, Epi-Liquidomics holds strong translational potential to refine cancer screening and advance minimally invasive precision oncology.

## Introduction

The global burden of cancer demands diagnostic strategies capable not only of detecting malignancy but of dynamically capturing tumor evolution, therapeutic adaptation, and minimal residual disease (Wong, J. et al., 2024; Pharo et al., 2025). Although tissue biopsy remains the clinical gold standard, its invasiveness, spatial sampling bias, and limited suitability for serial monitoring restrict its ability to represent tumor heterogeneity and temporal dynamics. These limitations have driven the development of liquid biopsy platforms that analyze circulating tumor-derived materials including cell-free DNA (cfDNA), extracellular vesicles (EVs), and nucleosomes for minimally invasive, longitudinal disease assessment (Wong D et al., 2024). To date, liquid biopsy has been largely mutation-centric, emphasizing circulating tumor DNA (ctDNA) genotyping. While effective for identifying actionable genomic alterations, mutation-based assays primarily detect stable clonal events and are constrained by low allelic fractions, variable tumor shedding, and the absence of driver mutations in certain malignancies (Wong J et al., 2024). Genomic profiling alone provides limited insight into tumor plasticity, microenvironmental adaptation, and reversible regulatory states governing therapy response and resistance. Expanding liquid biopsy beyond mutational landscapes toward tumor regulatory architecture is therefore essential.

We define Epi-Liquidomics as this conceptual expansion: a systems-level interrogation of circulating epigenetic information that encodes tumor regulatory state, evolutionary trajectory at the dispense of evolving technologies. Rather than a collection of assays, epi-liquidomics integrates DNA methylation and hydroxymethylation patterns, nucleosome positioning and chromatin fragmentation profiles, histone post-translational modifications, and non-coding RNA landscapes detectable in biofluids (Wong D et al., 2024; An et al., 2023; Gai and Sun, 2019). Operating at the interface of genomics and chromatin biology, it captures epigenetic signals released through cellular turnover and regulated secretion, generating a plasma-derived representation of tumor chromatin architecture and transcriptional activity unlike mutation-based assays (Cristiano et al., 2019; Fox-Fisher et al., 2023). Epigenetic signals provide multidimensional regulatory resolution. In the Circulating Cell-free Genome Atlas (CCGA) study, cfDNA methylation-based classifiers achieved >99% specificity with meaningful sensitivity across stages I–III cancers, demonstrating the feasibility of multi-cancer detection using epigenetic signatures (Liu et al., 2020). Importantly, methylation profiling enabled accurate tissue-of-origin localization, addressing a key limitation of mutation-centric liquid biopsy approaches. Subsequent clinical validation further confirmed the performance of a targeted methylation-based multi-cancer early detection assay in independent cohorts (Klein et al., 2021). Furthermore, Fragmentomics and nucleosome positioning analyses infer gene activity and cell-of-origin (Cristiano et al., 2019), while circulating nucleosomes bearing histone PTMs and EV-associated DNA and non-coding RNAs reflect active regulatory programs (Bai et al., 2024). A defining advantage of epi-liquidomics is its capacity to capture dynamic and reversible tumor states. Advances in next-generation sequencing, digital PCR, and machine-learning–based signal deconvolution are improving analytical sensitivity and scalability (Connal et al., 2023) and regulatory approval of methylation-based platforms demonstrates emerging clinical validity (Liu et al., 2020). In this Review, these multi-layered molecular and computational components collectively define the Epi-Liquidomics paradigm for precision oncology (Figure 1), expanding the scope of liquid biopsy beyond mutation detection toward dynamic mapping of tumor-state biology and establishing a workflow for temporally resolved precision oncology.

**FIGURE 1:**
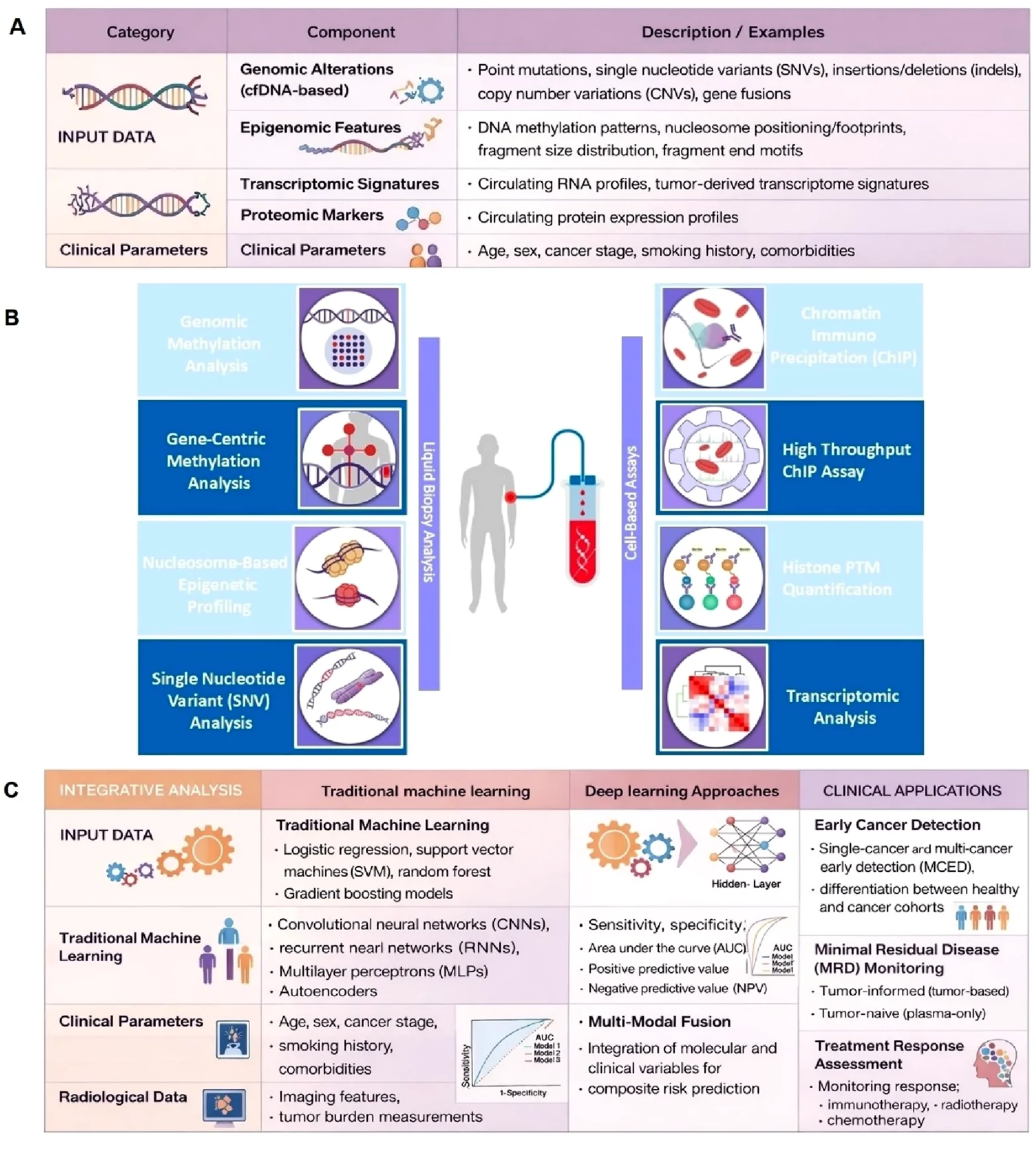
Epi-Liquidomics Framework and Integrative Computational Pipeline. **(A)** Multi-layer input data incorporated into Epi-Liquidomics, including cfDNA-derived genomic alterations (SNVs, indels, CNVs, gene fusions), epigenomic features (DNA methylation, nucleosome positioning, fragmentomics), circulating RNA signatures, proteomic markers, and clinical parameters. **(B)** Experimental workflow comprising cfDNA-based analyses (genome-wide and gene-centric methylation profiling, nucleosome mapping, SNV detection) and chromatin/cell-based assays (ChIP, high-throughput ChIP-seq, histone PTM quantification, transcriptomics). **(C)** Integrative computational architecture leveraging traditional machine learning and deep learning models for multi-modal data fusion to enable early cancer detection, minimal residual disease (MRD) monitoring, risk stratification, and treatment response assessment.

## Epigenetic mechanisms in cancer liquid biopsy

Epigenetic alterations detectable in liquid biopsy provide functional insight into tumor biology. DNA methylation, the addition of a methyl group to CpG cytosines is the most extensively validated circulating epigenetic biomarker. Hypermethylation of the SEPT9 promoter in plasma cell-free DNA (cfDNA) underlies a clinically approved colorectal cancer screening assay, demonstrating ∼68–79% sensitivity and ∼80–90% specificity for early-stage disease (Ørntoft et al., 2015; Loomans-Kropp et al., 2022). Aberrant cfDNA methylation of MGMT and RASSF1A has been correlated with glioma and lung cancer diagnosis and prognosis (Estival et al., 2019; Trombetta et al., 2025). Beyond 5-methylcytosine, 5-hydroxymethylcytosine (5hmC) profiling enhances resolution; in hepatocellular carcinoma, cfDNA 5hmC signatures distinguished cancer from cirrhosis and healthy controls with >85% sensitivity and specificity (Cai et al., 2019). Chromatin-derived signals further extend liquid epigenetic assessment. Circulating nucleosomes carrying tumor-associated histone marks including H3K27me3 and H3K9ac have been detected at elevated levels in lymphoma patients, correlating with disease severity and therapeutic response (Van Den Ackerveken et al., 2023). These findings suggest that circulating chromatin fragments reflect transcriptional activity and epigenetic state. Emerging adaptations of ChIP-seq now enable non-invasive mapping of histone modifications in plasma (Lotem et al., 2025). Circulating non-coding RNAs (ncRNAs) represent an additional regulatory layer. MicroRNAs and long non-coding RNAs remain stable in plasma due to encapsulation within extracellular vesicles or protein complexes. Elevated miR-21 levels consistently predict poor prognosis in breast, lung, and colorectal cancers (Song et al., 2013). Similarly, circulating HOTAIR has been associated with metastasis and reduced survival in breast cancer (Chen et al., 2021; Wang et al., 2022). These RNA signatures capture transcriptional and post-transcriptional dysregulation central to tumor progression. Technological advances have enabled high-sensitivity detection of these epigenetic markers. Bisulfite sequencing provides single-base methylation resolution from minimal cfDNA input (Lee et al., 2024), while hMe-Seal selectively enriches 5hmC-marked fragments. Droplet digital PCR permits ultrasensitive quantification of specific methylation events, supporting minimal residual disease detection (Wang et al., 2021). RNA sequencing and qRT-PCR enable robust ncRNA profiling (Chen et al., 2021). Biologically, epigenetic dysregulation is integral to oncogenesis. Hypermethylation-induced silencing of CDKN2A (p16) disrupts cell-cycle control (Warton and Samimi, 2015), whereas global hypomethylation promotes genomic instability and oncogene activation, including MYC (Trombetta et al., 2025). Epigenetic regulation of epithelial-to-mesenchymal transition (EMT), such as TWIST1 promoter methylation, modulates metastatic potential (Alfahed, 2023), and immune checkpoint pathways are similarly influenced by epigenetic remodeling. Clinically, cfDNA methylation assays enable early detection and multi-cancer screening, as demonstrated in the Circulating Cell-free Genome Atlas (CCGA) study, which achieved 67–88% sensitivity across multiple tumor types (Klein et al., 2018). Epigenetic markers also evolve during treatment: MGMT methylation dynamics correlate with temozolomide responsiveness in glioblastoma (Rosas-Alonso et al., 2024), and circulating miRNA shifts have been linked to chemoresistance in breast cancer (Cardinali et al., 2022). Together, circulating epigenetic profiling integrates mechanistic insight with clinical application, reinforcing its central role in precision oncology.

### Technological platforms enabling epi-liquidomics

Technological platforms enabling epi-liquidomics have undergone substantial advancements by 2025, integrating high-resolution and multi-dimensional profiling capabilities that leverage the latest molecular biology and computational techniques. Methylome profiling is foundational, with whole-genome bisulfite sequencing (WGBS) providing single-base resolution of 5-methylcytosine modifications but constrained by high DNA input requirements and potential for DNA damage during bisulfite conversion. To address sensitivity limitations, cfMeDIP-seq (cell-free methylated DNA immunoprecipitation sequencing) has emerged as a complementary enrichment-based approach, enabling efficient detection of methylated cfDNA fragments from low-input plasma samples with reduced degradation (Baca et al., 2023; Liu et al., 2020). Targeted methylation panels applying capture-based or PCR enrichment technologies prioritize clinically relevant CpG regions, supporting scalable, cost-effective diagnostics though at the expense of genome-wide discovery (Baca et al., 2023).​ Histone modification detection technologies have evolved to incorporate mass spectrometry-based proteomics for detailed quantification of circulating histone post-translational modifications (PTMs), revealing epigenetic regulatory landscapes reflective of tumor biology. Innovations in chromatin immunoprecipitation assays adapted for low abundance plasma nucleosomes, including CUT&Tag and CUT&RUN, now permit precise mapping of histone marks at regulatory elements in liquid biopsy samples, providing functional readouts of gene regulation inaccessible to mutation-based methods (Ren et al., 2024; Baca et al., 2023).​ Chromatin fragmentomics exploits the analysis of cfDNA fragment size distributions, end motifs, and nucleosome positioning signatures to enhance tissue-of-origin inference, marking a breakthrough in non-invasive cancer typing. Recent studies demonstrate that fragmentomics yields orthogonal data to methylation profiling, together producing refined cancer detection and localization (Cristiano et al., 2019; Ulz et al., 2019).​ Parallel advances in RNA epigenetics and small RNA profiling harness next-generation sequencing of circulating miRNAs and exosomal RNA cargo, uncovering RNA modifications and regulatory RNA species linked to tumor progression. Single-vesicle analytical platforms now enable high-resolution characterization of extracellular vesicle heterogeneity, supporting biomarker specificity and mechanistic insights (Ren et al., 2024).​

Machine learning integration underpins the interpretation of complex multiomic data streams, with deep learning algorithms excelling in pattern recognition within cfDNA methylation landscapes and fusion models combining epigenomic, transcriptomic, and proteomic data. These AI-powered frameworks advance biomarker discovery and clinical decision support, promoting precision oncology through actionable, interpretable diagnostics (Lau et al., 2023; Wong J et al., 2024).​ Together, these technological advances construct a sophisticated, multi-layered platform for epi-liquidomics that is poised to redefine early cancer detection, prognosis, and adaptive treatment monitoring in clinical practice (Figure 2).

**FIGURE 2:**
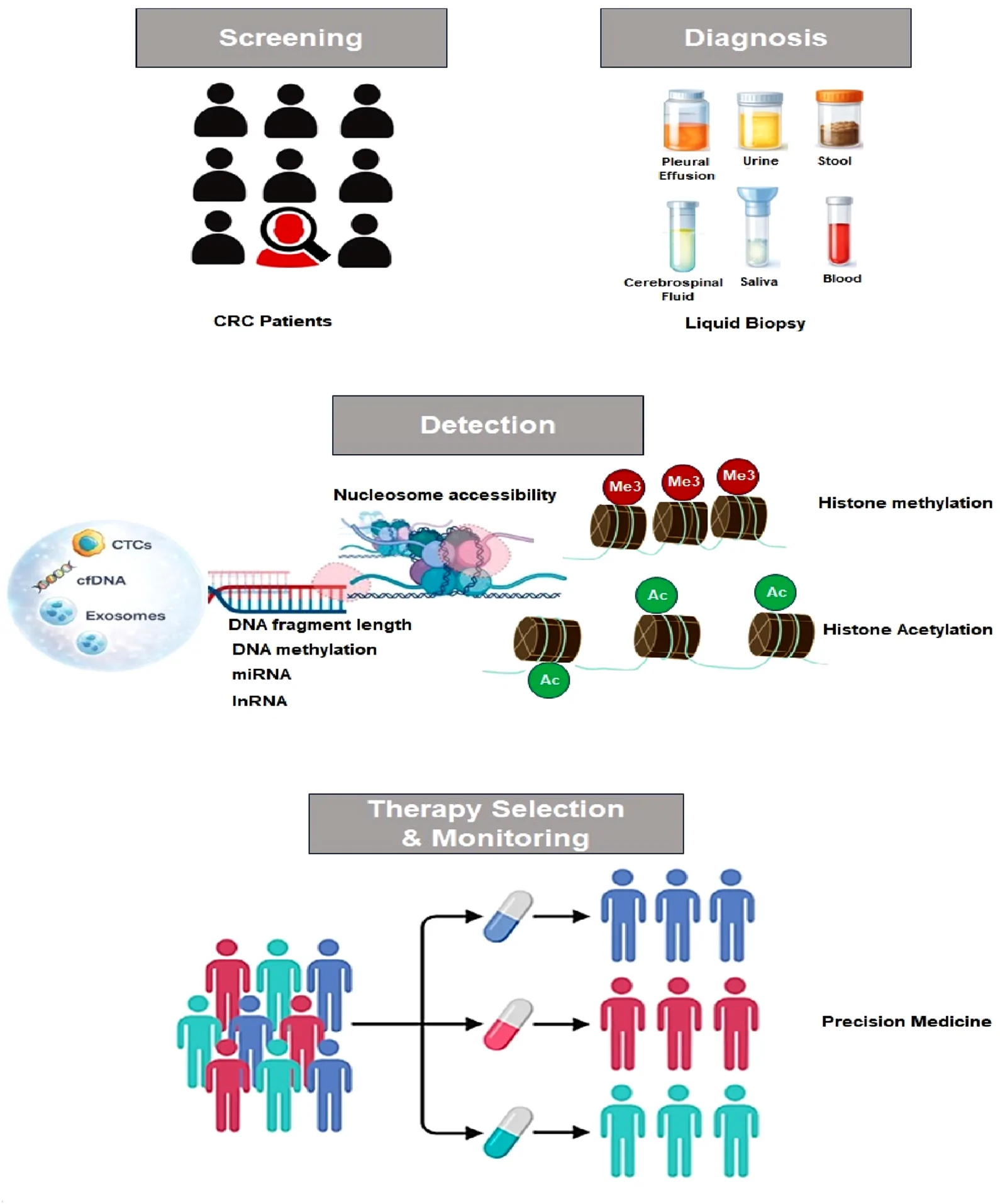
Epi-Liquidomics: From Population Screening to Precision Oncology Clinical workflow of Epi-Liquidomics across the cancer care continuum. Various biofluids—including blood, urine, stool, saliva, cerebrospinal fluid, and pleural effusion—serve as sources of circulating tumor-derived analytes such as cfDNA, circulating tumor cells (CTCs), and extracellular vesicles. Epigenetic signatures including DNA methylation, nucleosome accessibility, histone modifications (methylation and acetylation), and non-coding RNA profiles enable early cancer detection, risk stratification, therapy selection, and longitudinal disease monitoring. This paradigm positions Epi-Liquidomics as a transformative strategy for non-invasive precision oncology.

### Applications in cancer precision medicine

Epigenetic biomarkers in liquid biopsy enable sensitive, detection of tumor-associated molecular alterations. Methylation of the *SEPT9* promoter in plasma cfDNA, the basis of the FDA-approved Epi proColon® test, represents a clinically validated approach for colorectal cancer screening with strong performance in early-stage disease (Shirley, 2020). In lung cancer, methylation of genes such as *SHOX2* and *RASSF1A* distinguishes patients from high-risk controls with >80% accuracy (Liu B et al., 2024; Zhang et al., 2024), while in breast cancer, circulating tumor suppressor gene methylation and oncogenic microRNAs including miR-21 support early detection and risk assessment (Mihai et al., 2024). Genome-wide cfDNA methylation platforms evaluated in the CCGA study further demonstrate multi-cancer detection capability across malignancies such as pancreatic and ovarian cancer (Klein et al., 2021). Beyond detection, circulating epigenetic signatures provide clinically actionable prognostic and predictive information. MGMT promoter methylation in cfDNA predicts response to alkylating agents in glioblastoma (Szylberg et al., 2022). Plasma-based methylation panels, including *NDRG4* and *BMP3* in colorectal cancer, correlate with tumor stage, recurrence risk, and overall survival (Chen et al., 2019). Integrating cfDNA methylation signatures with genomic alterations, such as *EGFR* mutations in lung cancer, enhances risk stratification and therapeutic decision-making (Liu G et al., 2024). Epigenetic liquid biopsy also provides a dynamic measure of treatment adaptation. Reductions in *RASSF1A* methylation during targeted therapy correlate with tumor regression, often preceding radiographic changes (Gao et al., 2022). Re-emergence of methylated cfDNA fragments enables early detection of minimal residual disease and relapse (Colmenares et al., 2022). Altered MGMT methylation patterns signal temozolomide resistance in glioblastoma, while elevated circulating miR-155 and miR-21 levels are associated with chemotherapy resistance in breast cancer (Chen et al., 2019; Pandey et al., 2024). Importantly, emerging Epi-Liquidomics research extends beyond biomarker detection to resolving tumor heterogeneity and lineage plasticity. In small cell lung cancer (SCLC), cfDNA methylation profiling identifies clinically relevant transcription factor driven subtypes ASCL1-, NEUROD1-, POU2F3-, and YAP1-defined states enabling molecular stratification in a highly heterogeneous and therapy resistant malignancy (Heeke et al., 2024). Furthermore, epigenetic interrogation of cfDNA may capture signatures associated with extrachromosomal DNA (ecDNA) amplification, a key driver of oncogene overexpression and tumor evolution. Given the central role of ecDNA in aggressive tumor behavior and therapeutic resistance (Pongor et al., 2023), Epi-Liquidomics holds potential for tracking dynamic oncogene amplification states and adaptive resistance mechanisms. Representative recent DNA methylation–based liquid biopsy studies across multiple cancer types, including early-stage detection settings, are summarized in Table 1.

**TABLE 1:** Summary of recent DNA methylation–based cancer liquid biopsy studies.

Study (reference)	Cancer type(s)	Sample size (N)	Marker(s)/Assay	Clinical application/Findings
Barault et al. (2015)	GBM/mCRC	164/83	MGMT (bisulfite dPCR)	Prognosis; predicting therapy response
Barault et al. (2017)	mCRC	182	EYA4, GRIA4, ITGA4, MAP3K14-AS1, MSC (bisulfite dPCR)	Predicting therapy response
Chimonidou et al. (2012)	Breast cancer (BC)	114	SOX17 (MSP)	Diagnosis: Sensitivity 38%, specificity 98%
Fackler et al. (2014)	Metastatic BC (mBC)	57	Panel of 10 markers (multiplexed MSP)	Diagnosis: 91% sensitivity/96% specificity
Gordevičius et al. (2018)	Prostate cancer	33	Multiple markers (microarray)	Predicting therapy response
Henriksen et al. (2016)	Pancreatic cancer (PC)	95	Panel of 28 markers (MSP)	Tumor staging
Herbst et al. (2017)	Metastatic colorectal cancer (mCRC)	467	HPP1 (MSP)	Prognosis and response prediction
Hulbert et al. (2016)	Non-small cell lung cancer (NSCLC)	150	SOX17, TAC1, HOXA7, CDO1, HOXA9, ZFP42 (MSP)	Diagnosis: 93% sensitivity/62% specificity
Melson et al. (2013)	CRC/PC	30/30	CYCD2, HIC, VHL, MYF3, TMS, GPC3, SRBC (MSP)	Diagnosis: CRC 83%/94%; PC 81%/67%
Potter et al. (2014)	CRC	50	SEPT9 (MSP)	Diagnosis: 68% sensitivity/79% specificity
Schröck et al. (2017)	HNSCC	425	SHOX2, SEPT9 (MSP)	Diagnosis: 52% sensitivity/95% specificity; prognostic significance
Wen et al. (2015)	Hepatocellular carcinoma (HCC)	36	Multiple markers (MCTA-seq)	Diagnosis: 94% sensitivity/89% specificity
Widschwendter et al. (2017a)	Breast cancer (BC)	925	EFC (BS-seq)	Prognostic indication
Widschwendter et al. (2017b)	Ovarian cancer (OC)	151	Panel of 3 markers (BS-seq)	Diagnosis: 41% sensitivity/91% specificity
Xu et al. (2017)	HCC	1,098	Multiple markers (BS-seq)	Diagnosis: 83% sensitivity/91% specificity
Song et al. (2017)	Colorectal cancer	1133	SEPT9 promoter methylation (epi proColon®)	Early detection and screening; sensitivity 68–79% specificity 80–90%
Liu J et al. (2024)	Lung cancer	240	SHOX2 and RASSF1A methylation panel (quantitative MSP)	Diagnosis and monitoring; diagnostic accuracy >80%
Cai et al. (2021)	Hepatocellular carcinoma	135	Global 5-hmC profiling in cfDNA	Early detection; sensitivity and specificity >92%
Klein et al. (2021)	Multi-cancer (plasma cfDNA)	2,823	Targeted cfDNA methylation + ML classifier (MCED)	Cancer signal detection + tissue-of-origin prediction; specificity 99.5%, sensitivity 51.5% overall

### Comparison with genomic liquid biopsy approaches

Epi-Liquidomics technologies demonstrate superior analytical performance over genomic assays, with sensitivity and specificity profiles that are increasingly robust and clinically relevant in early cancer detection Table 2. Unlike mutation-based liquid biopsy approaches, which depend on sufficient tumor DNA shedding and may exhibit limited sensitivity in early-stage disease, methylation profiling captures widespread epigenomic reprogramming and has demonstrated improved performance in tumors with low mutational burden (Liu et al., 2020; Liu, 2024). Fragmentomic and nucleosome-based approaches further extend detection beyond mutation tracking by interrogating chromatin organization and tissue-of-origin signals (Cristiano et al., 2019). Recent studies report methylation-based cfDNA assays achieving sensitivities ranging from approximately 67% to over 90%, depending on cancer type and stage, with specificities frequently above 90%, thereby minimizing false positives in a screening context (Han et al., 2025; Liu M et al., 2020; Ferrier et al., 2024). For instance, lung cancer early detection methylation panels combining SHOX2 and PTGER4 markers have shown sensitivities of ∼67% at 90% specificity, whereas larger multi-gene methylation models achieve sensitivities approaching 85–95% with specificities of 90–97%, substantially outperforming mutation-centric assays at comparable early stages (Han et al., 2025; Wong D et al., 2024).​ Regarding tissue-of-origin accuracy, methylation signatures provide hierarchical and tissue-specific patterns that enable accurate localization of tumor origin in over 80% of cases across multiple cancer types (Cristiano et al., 2019; Liu M et al., 2020). This contrasts with mutation-based approaches, which often lack sufficient discriminatory power due to shared or ubiquitous mutations. Additionally, epigenetic profiles uniquely capture components of the tumor microenvironment, including immune and stromal cell infiltration signatures deduced from cfDNA methylation patterns, providing prognostic and predictive insight otherwise inaccessible through genomic assays alone (Zhou et al., 2025; Ren et al., 2024).​ Cost-effectiveness analyses suggest epigenetic liquid biopsy tests can be more economically scalable than broad-panel NGS mutation assays, attributed to targeted enrichment of methylation loci and methylation-specific PCR techniques that reduce sequencing depths and bioinformatic complexity (Baca et al., 2023; Liu et al., 2020). Thus, epigenetic liquid biopsy represents a promising analytical framework balancing heightened diagnostic power, biological relevance, and clinical feasibility for precision oncology as compared to genomic liquid biopsy (Table 3).

**TABLE 2:** Comparative overview of epi-liquidomics and genomic liquid biopsy.

Feature	Epi-liquidomics (epigenetic profiling)	Genomic liquid biopsy (mutation-based)
Primary biomarkers	DNA methylation patterns, nucleosome positioning, chromatin fragmentation, non-coding RNAs	Somatic mutations, copy number variations, gene fusions
Biological signal type	Regulatory state of tumor cells (epigenetic reprogramming)	Genetic alterations (sequence-level changes)
Early-stage detection potential	High specificity; methylation changes often occur early in tumorigenesis	Limited sensitivity in early-stage cancers due to low mutation burden
Tissue-of-origin prediction	Strong tissue specificity of methylation signatures enables accurate localization	Often limited; requires mutation context or additional modeling
Tumor heterogeneity capture	Reflects transcriptional states, subtype plasticity, and regulatory evolution	Captures clonal mutations but may miss epigenetic plasticity
Minimal residual disease (MRD)	Potentially sensitive through methylation-based residual signal detection	Established in several cancers but dependent on known mutations
Dynamic monitoring	Tracks epigenetic reprogramming and therapy-induced regulatory shifts	Tracks emergence of resistant mutation clones
Clinical maturity	Emerging; limited regulatory approvals	Clinically validated platforms with regulatory clearance
Technical challenges	Bisulfite conversion variability, bioinformatic complexity, standardization issues	Low allele fraction detection, background noise, clonal hematopoiesis
Current limitations	False positives in screening settings; cost and workflow harmonization needed	Limited tissue-of-origin resolution; may miss non-mutational drivers

**TABLE 3:** Epigenetic liquid biopsy studies in translational and clinical trial phases.

Platform/Trial	Cancer type	Biomarkers tested	Sample size	Key findings	Clinical status/Outcome
OvaPrint	High-grade serous ovarian cancer	cfDNA methylation (unpublished proprietary panel)	Not specified	Developed a cfDNA methylation-based liquid biopsy for risk assessment	Early-stage study; findings reported in clinical cancer research; Buckley et al., 2023
EarlyTect® L	Lung cancer	PCDHGA12 methylation (serum)	522 participants	Sensitivity 77.8%, specificity 92.3%	Validated epigenetic biomarker test for lung cancer; Rendek et al., 2024
Epi proColon® 2.0 CE/HCCBloodTest	Hepatocellular carcinoma	Methylation (epi proColon panel)	Cirrhosis cohort: 98 HCC out of 289	Sensitivity 90.6%, specificity 87.2%—better than AFP biomarker	Ongoing prospective trial NCT03311152
Epi proColon®/Epi proLung/Lung EpiCheck	Colorectal and lung cancer	Methylation markers (SEPT9; SHOX2, PTGER4; 6-gene panel)	CRC: Multiple studies; LC: 118–172 patients	Epi proColon: FDA-approved; epi proLung: Sensitivity 67%, specificity 90%; lung EpiCheck: Early LC detection up to 85% sensitivity, specificity ∼64%	Epi proColon (FDA-approved), epi proLung/EpiCheck NCT03218423
PanSeer (Taizhou longitudinal study)	Pan-cancer (multiple types)	ctDNA methylation (11,787 CpGs)	605 analyzed from 123,115 cohort	Detection rate 88% in diagnosed patients; specificity 96%; 95% detection in asymptomatic individuals later diagnosed	Longitudinal cohort study supporting early detection potential; Chen et al., 2020
GRAIL (Galleri™)	Pan-cancer	NGS methylation panel (∼1.1M CpGs)	4,077	Multi-cancer early detection (MCED); sensitivity 51.5%, specificity 99.5%	GRAIL (Galleri™), GRAIL (2021)
Guardant Reveal™	CRC/Breast cancer	NGS, 500 genes and 4 Mb DMRs	84 (CRC); 20 (breast)	MRD detection; CRC sens. 55.6%, breast sens. 85%, both spec. 100%	Guardant Reveal™ Guardant Reveal™ (2025)
ADELA (cfMeDIP-seq)	Pan-cancer	Whole-genome methylome (cfMeDIP-seq)	MCED: 4322; HNSCC: 93; RCC: 151	MCED AUROC 0.94; prognosis: HNSCC recurrence HR 3.51; RCC recurrence HR 13.28	ADELA (cfMeDIP-seq); Liu G et al., 2024
Esophageal cancer methylation trial	Esophageal cancer (EC)	qMSP methylation (Epo, MT-1A, PDGFRA, HOXB13, TRIM15, SEPT9, etc.)	Diagnostic cohort: 609 EC	Diagnostic efficacy assessed via sensitivity, specificity, AUC in large multicenter trial	Clinical trial (ChiCTR2400083525); results pending
IvyGene Dx Liver cancer test	Hepatocellular carcinoma	cfDNA methylation (NGS)	Not specified here	Sensitivity 95%, specificity 97.5%	Early-stage; received regulatory breakthrough Device designation; trial NCT03694600 ongoing
Carcimun®	Multi-cancer (differentiating cancer vs. inflammation)	cfDNA-based diagnostic assay	Not specified here	90.6% accuracy for cancer vs. inflammation; 95.4% overall accuracy; 98.2% discrimination performance	Early-stage detection; cancer vs. inflammation discrimination Walter et al. (2025)

### Clinical translation: challenges and opportunities

The clinical implementation of epigenetic liquid biopsy profiling faces technical, biological, computational and regulatory challenges that affect sensitivity, reproducibility, interpretation, and integration into patient care. Tumor derived epigenetic biomarkers, such as methylated cfDNA fragments, are often present at very low frequencies in early-stage disease or minimal residual disease (MRD), complicating reliable detection. Even digital PCR and next-generation sequencing may struggle to distinguish tumor-derived signals from background cfDNA originating from normal cells (Wong J et al., 2024). Specificity is further compromised by biological confounders, including clonal hematopoiesis, inflammation and benign methylation alterations that can mimic cancer associated patterns, increasing false-positive risk (Liu J et al., 2024). Pre-analytical and analytical variability further limits reproducibility. Differences in blood collection tubes, processing times, centrifugation, DNA extraction, and bisulfite conversion can significantly alter epigenetic profiles (Pandey et al., 2024; Veronese et al., 2025). Delayed plasma separation may cause leukocyte lysis and genomic DNA contamination, diluting tumor-derived signals. CfDNA fragmentation patterns (“fragmentomics”) also influence assay performance and require harmonized handling (Wong D et al., 2024). Though, international consortia such as ELBS and BloodPAC aim to standardize workflows and improve cross-study reproducibility. Further, bioinformatic complexity presents additional barriers to its translation. Large-scale methylation datasets require robust pipelines for quality control, alignment, methylation calling and extraction of cancer-specific signatures from heterogeneous data (Morrison-Smith et al., 2022). Limited population-scale reference methylomes constrain classification accuracy and integration with multi-omic and clinical variables depends on machine learning frameworks that remain under refinement (Wong J et al., 2024). Regulatory approval and cost effectiveness also remain as a challenge. While mutation-based platforms such as Guardant360 CDx and FoundationOne Liquid CDx have achieved regulatory clearance, epigenetic assays are still emerging (Wong J et al., 2024). Biological variability further constrains performance. Tumor shedding varies by size, anatomical location, blood–brain barrier constraints and treatment status (Berchuck et al., 2025; Coppola et al., 2025), and intra- and inter-tumoral heterogeneity may obscure clinically relevant subclones, reinforcing the need for multimodal panels and longitudinal sampling (Coppola et al., 2025; Veronese et al., 2025). However, a prospective screening data underscore both feasibility and limitations. In the PATHFINDER study, a methylation-based multi-cancer early detection assay demonstrated clinical feasibility; however, only 38% of positive tests were confirmed cancers, while 62% were false positives, highlighting challenges in positive predictive value and downstream diagnostic burden (Schrag et al., 2023). Key preclinical and clinical validation studies supporting the role of Epi-Liquidomics in early cancer detection and precision oncology are summarized in Table 3.

## Future perspectives and emerging technologies

The future of epigenetic liquid biopsy lies in multi-omics integration, combining epigenomic, genomic and transcriptomic data to generate a comprehensive molecular profile of tumors. Integrating cfDNA methylation with mutation and circulating RNA signatures has improved early detection accuracy in lung and colorectal cancers compared with single-analyte approaches (Nguyen et al., 2025). Advances in next-generation sequencing including long-read and single-cell platforms enhance detection of complex structural and epigenetic variation. Nanopore sequencing enables direct, amplification-free, single-molecule analysis, simultaneously identifying genetic mutations and epigenetic marks such as 5mC and 5hmC in their native context. Pilot studies in pancreatic and brain cancers demonstrate sensitive and cost-effective detection of rare epigenetic signatures (Halliwell et al., 2025; Song et al., 2025; Shuai et al., 2023). CRISPR-based systems further expand precision diagnostics; CRISPR-Cas12 has been used to detect colorectal cancer–specific methylation patterns, highlighting its screening potential (Tian et al., 2025). Nanotechnology platforms, including magnetic nanoparticles, improve isolation of rare biomarkers such as ctDNA and exosomes, enhancing profiling sensitivity (Bashir et al., 2024). Integration with artificial intelligence (AI) and machine learning (ML) facilitates biomarker discovery and interpretation of high-dimensional datasets; AI-driven models have identified methylation signatures predictive of treatment response and resistance in breast and lung cancers, outperforming conventional statistical approaches (Sahoo et al., 2025; Khan et al., 2026; Danishuddin et al., 2025). Large prospective studies, including the PATHFINDER trial, are evaluating multi-cancer cfDNA methylation assays for early detection in asymptomatic populations (Guerra et al., 2023). Beyond oncology, disease-specific cfDNA methylation profiling is also being explored in autoimmune disorders, neurodegenerative diseases, and prenatal diagnostics, underscoring the broader translational potential of epigenetic liquid biopsy (Veronese et al., 2025). Collectively, multi-omics integration, advanced sequencing technologies, AI-enabled analytics and expanded clinical applications are positioning epi-liquidomics as a scalable platform for early detection, longitudinal monitoring, and personalized intervention.

## Conclusion

In summary, liquid biopsy–based epigenetic profiling provides a real time monitoring of tumor regulatory states. Analysis of circulating DNA methylation, histone modifications, and non-coding RNA signatures enables early detection, monitoring of tumor evolution, and identification of therapeutic resistance. Unlike static tissue biopsies, these approaches capture systemic tumor dynamics and more accurately reflect clonal selection and epigenetic reprogramming over time. Clinical integration has the potential to enhance risk stratification, minimal residual disease detection, and treatment optimization, particularly in heterogeneous malignancies. Broader implementation requires improved analytical sensitivity and clinical validation. Advances in sequencing technologies and AI-enabled multiomic integration are expected to enhance assay precision and scalability. Large-scale prospective validation and integration with established clinical workflows remain necessary before widespread adoption.
